# The development of the revised COPE 68 inventory with English and Slovak versions

**DOI:** 10.3389/fpsyg.2023.1202571

**Published:** 2023-06-29

**Authors:** Júlia Halamová, Martin Kanovský, Bronislava Strnádelová, Martina Baránková, Katarína Greškovičová

**Affiliations:** ^1^Institute of Applied Psychology, Faculty of Social and Economic Sciences, Comenius University in Bratislava, Bratislava, Slovakia; ^2^Institute of Social Anthropology, Faculty of Social and Economic Sciences, Comenius University in Bratislava, Bratislava, Slovakia

**Keywords:** coping, COPE inventory, ESEM, factor analysis, reliability

## Abstract

**Introduction:**

Although there have been several attempts at improving the COPE Inventory, the factor structure of the instrument is still in dispute. In addition, studies have shown low reliability coefficients for some of the first-order factors, with Mental Disengagement having the lowest factor loadings. In a recent study on the external validation of the instrument, two additional first-order factors were identified in the qualitative analysis, namely *Self-care* and *Care for Others*.

**Methods:**

Based on these arguments we created the Revised COPE 68 Inventory, changing some of the problematic items in the first order factor *Mental Disengagement* and adding items for the two new factors (*Self-care* and *Care for Others*). We then tested its reliability and performed factor analyses on the first and second-order factorial structure. The data were collected through social media in two languages, English and Slovak, using convenience and snowball sampling techniques. The English sample contained 834 participants with mean age 25.27  years (SD = 8.467) and the Slovak sample comprised 1,425 participants with mean age 33 years (SD = 14.59). For the statistical analyses we used Exploratory Structural Equation Model (ESEM) analyses with target rotation and WLSMV, Exploratory and second-order confirmatory factor analysis with the scores of the COPE Inventory and EFA.

**Results:**

The Revised COPE 68 inventory had a good fit for all 17 first-order factors in both languages, including for the new factors *Self-care* and *Care for Others*. It appears that the first-order factors form a three-factor solution in both samples, consisting of active coping, social–emotional coping and avoidant coping. The revised *Mental Disengagement* has better psychometric properties as well.

**Discussion:**

The Revised COPE 68 inventory was found to be a reliable multidimensional instrument for measuring various coping strategies in both the English and Slovak language versions.

## 1. The COPE inventory

The COPE Inventory (Carver et al., 1989) is the most frequently used instrument for measuring coping (Kato, 2015) as it measures a variety of functional and dysfunctional coping strategies. Carver (2013a) defines coping as “efforts to prevent or diminish threat, harm, and loss, or to reduce the distress.” The advantage of this inventory is that it was constructed based on Lazarus and Folkman’s (1984) transactional model of stress and coping and Carver and Scheier’s (1981) behavioural self-regulation model, although not empirically so (cf. Folkman and Lazarus, 1985; McCrae and Costa, 1986).

The measure has been theoretically constructed so as to contain 14 conceptually distinct first-order factors that were confirmed through a factor analysis (Carver et al., 1989). An additional subscale *Humor* was added afterwards (Deisinger et al., 1996). Thus, the COPE Inventory (Carver et al., 1989) consists of 15 first-order factors with each factor containing four items, 60 items altogether. The first-order factors in the inventory are as follows: 1. *Acceptance*; 2. *Active Coping*; 3. *Behavioural Disengagement*; 4. *Denial*; 5. *Use of Emotional Support*; 6. *Humor*; 7. *Use of Instrumental Support*; 8. *Mental Disengagement/Self-distraction*; 9. *Planning*; 10. *Positive Reinterpretation*; 11. *Religion*; 12. *Restraint*; 13. *Substance Use*; 14. *Suppression of Competing Activities*; and 15. *Focus on and Venting of Emotions*.

The COPE comes in a long 60-item version with 15 distinct coping strategies (described above) and a brief 28-item version with 14 distinct coping strategies. The instruments can be used to tap either dispositional or situational coping strategies. Studies have shown that the brief version is equivalent to the long one. Carver (1997) reported a remarkably similar factor structure with good estimation of internal reliability.

The English-language inventory (Carver et al., 1989) has been translated into various languages and published in, e.g., Chinese (Hsu, 2003), Croatian (Hudek-Knežević et al., 1999), Estonian (Kallasmaa and Pulver, 2000), French (Desbiens and Fillion, 2007), Italian (Bongelli et al., 2022), Polish (Juczyňski and Ogiňska-Bulik, 2012), Portuguese (Brasileiro et al., 2016), Romanian (Crașovan and Sava, 2013), Russian (Garanyan and Ivanov, 2010), Saudi (Alghamdi, 2020), Slovak (Halamová et al., 2022), Spanish (Perczek et al., 2000), Turkish (Şahan and Karademir, 2022), and Vietnamese (Matsumoto et al., 2020).

### 1.1. The development of the revised COPE 68 inventory

In spite of the work performed by Carver et al. (1989) and Carver (1997) to improve the coping instrument, the factor structure of the COPE inventories is still disputed (Bose et al., 2015; Brasileiro et al., 2016; Solberg et al., 2021). Several studies have reported ambiguous results on number and characteristics of the first-order factors. According to a systematic review by Solberg et al. (2021) the number of factors reported in studies ranges from 2 to 15, with a two-factor structure being most frequent.

The most problematic factor appears to be mental disengagement coping strategy (e.g., Carver, 1997; Kallasmaa and Pulver, 2000; Garanyan and Ivanov, 2010; Crașovan and Sava, 2013). A recent study by Halamová et al. (2022) confirmed that mental disengagement is problematic. Their study reported low reliability coefficients and low factor loadings.

Halamová et al. (2022) externally validated the COPE inventory (Carver et al., 1989) and proposed that there were two additional first-order factors that were missing from the original scale. They are *Self-care* and *Care for Others*, which are coping strategies individuals use to reduce distress and the risk of harm and loss related to stressful experiences, which is similar to the way in which the original 15 coping strategies work (Carver, 2013b).

Regarding higher-order factors, in their original study Carver et al. (1989) identified four second-order factors: 1. problem-focused coping (including active coping, planning and suppressing competing activities), 2. emotion-focused coping (consisting of seeking instrumental social support, seeking emotional social support and venting), 3. disengagement (involving denial, mental disengagement and behavioural disengagement), and 4. acceptance (linked to acceptance, restraint coping and positive reinterpretation). Nevertheless, most subsequent studies identified variations in the higher-order factor structure of the COPE Inventory, ranging from three (Stowell et al., 2001; Litman, 2006) to five factors (Sica et al., 1997). A recent study (Solberg et al., 2021) reviewed the higher-order factor structure and reported on the most common inner structures. Where there were two higher-order factors, the most frequently mentioned were approach and avoidant coping. And where there were three higher-order factors, they tended to be disengaged, active and social support coping. Solberg et al. (2021) discussed the possible reasons for the variation in the number of higher-order factors and suggested that situational or dispositional coping and the language version could be the source. Consequently, it is important to analyze multiple language versions at the same time and test the differences. That led us to collect data on the English and Slovak versions of the instrument.

## 2. The aim of the current study

Our decision to develop the Revised COPE 68 inventory was based on the problems with the factor solution in the COPE 60 item version and on previous findings indicating there were two additional factors (Halamová et al., 2022). Several studies have shown low reliability coefficients for the first-order factors, with *Mental Disengagement* having the lowest factor loadings (Carver et al., 1989; Garanyan and Ivanov, 2010; Crașovan and Sava, 2013). In addition, the solution for the first-order structure is inconsistent, ranging from 2 to 15 higher-order factors (Solberg et al., 2021). In a recent study on the external validation of the instrument, two additional first-order factors were identified in the qualitative analysis (Halamová et al., 2022), *Self-care* and *Care for Others*. Lastly, the higher-order factorial structure is inconsistent, ranging from three (Stowell et al., 2001; Litman, 2006) to five second-order factors (Sica et al., 1997). Based on these arguments we decided to create the Revised COPE 68 Inventory by changing the items relating to the most problematic first-order factor, *Mental Disengagement*, and adding items for the two new factors (*Self-care* and *Care for Others*). We also wanted to test its reliability and perform factor analyses on both the first-order structure and second-order structure.

## 3. Methods

### 3.1. Procedure

We put together a battery of sociodemographic questions (sex, age, education, family status, and employment status) and the Revised COPE Inventory. There were two language versions of the battery, English and Slovak. The online data gathering was disseminated through social media using convenience and snowball sampling techniques. Data were collected in accordance with the ethical standards of the institutional and/or national research committee and in accordance with the 1964 Helsinki Declaration and its later amendments or comparable ethical standards. The study protocol was approved by the Ethical Committee of the Faculty of Social and Economic Sciences at Comenius University, Bratislava.

#### 3.1.1. Research sample 1 – English

Our English sample contained 834 participants (508 were women, 304 were men and 22 were other). The mean age was 25.27 years (SD = 8.467) and the ages ranged from 18 to 73 years. Participants were not exclusively English native speakers, they were just English speakers. The whole battery was in English and included an informed consent form at the beginning of the survey.

#### 3.1.2. Research sample 2 – Slovak

Our Slovak sample comprised 1,425 participants (964 were women, 452 were men and 9 were other). The mean age was 33 years (SD = 14.59) and the ages ranged from 18 to 99 years. Participants were not exclusively Slovak native speakers, they were just Slovak speakers. The whole battery was in Slovak and included an informed consent form at the beginning of the survey.

### 3.2. Measures

#### 3.2.1. The revised COPE 68 inventory

Besides finding *Mental Disengagement* had low reliability and low factor loadings, Halamová et al. (2022), suggested that items 16, 31, and 43 should be reformulated given the differences in degree of specificity in the disengaging activities. For example, item number 31 states: “*I go to movies or watch TV to think about it less*.” Based on their results, Halamová et al. (2022) concluded that this statement could be misinterpreted as being a relaxation technique rather than an activity for disengaging. Moreover, these days people often check their mobile phones rather than watch TV. It appeared that the well-formulated items (e.g., item no. 2 “*I turn to work or other substitute activities to take my mind off things*.”) are the ones that are more general and do not specify activities that people use to disengage. Therefore, in an expert panel discussion the authors created new, more generally formulated items for *Mental Disengagement*. In addition, we elaborated new items for *Self-care* and *Care for Others* (4 items for each coping strategy) to reflect participants’ assertions in the external validation research study by Halamová et al. (2022). See Appendix 2 for the final wording. Afterwards, we randomized the order of all 68 items. For the items in the English language version of the Revised COPE 68 Inventory see Appendix 3, and for the Slovak version see Appendix 4.

In this study we used the Revised COPE 68 Inventory (Carver et al., 1989), comprised of 68 items and 17 first-order factors with each factor containing four items. Participants responded using a 4-point Likert scale (1 = I usually do not do this at all; 4 = I usually do this a lot). The first-order factors in the inventory are as follows: 1. *Acceptance* – being accepting of the situation; 2. *Active Coping* – performing specific actions to deal with the situation; 3. *Behavioural Disengagement* – reactive refusal to deal with stress; 4. *Denial* – denying the reality of the situation; 5. *Use of Emotional Support* – relying on others for empathy and understanding; 6. *Humor* – joking about the situation; 7. *Use of Instrumental support* – seeking instrumental help from others, such as advice or information; 8. *Mental Disengagement/Self-distraction* – doing activities that distracts one from unpleasant thoughts related to the problem; 9. *Planning* – strategizing how to deal with a stressful situation; 10. *Positive Reinterpretation* – finding positives in a stressful situation; 11. *Religion* – using religious activities to cope, such as praying; 12. *Restraint* – making sure one does not respond to stress in a reactive way; 13. *Substance Use* – using substances to deal with a distressing situation; 14. *Suppression of Competing Activities* – intentionally avoiding activities that do not help the person deal with the problem; 15. *Focus on and venting of emotions* – sharing negative emotions; 16. *Self-care* – managing stress levels by engaging in pleasant and unpleasant activities and fulfilling needs; and 17. *Care for Others* – helping others to relieve their stress to help oneself relieve one’s stress (Halamová et al., 2022).

### 3.3. Data analysis

We used Mplus version 8.4 for the statistical analysis (Muthén and Muthén, 2017). Specifically, we used Exploratory Structural Equation Model (ESEM) analyses with target rotation with WLSMV, second-order confirmatory factor analysis (CFA) and Exploratory Factor Analysis (EFA) with the COPE Inventory’s scores. As Asparouhov and Muthen (2009) noted, performing a full-fledged CFA is risky when the factor structure is not known or is uncertain. In these cases it is best to perform an ESEM with target rotation specifying the theory-driven loadings while permitting small cross-loadings. We will perform the ESEM model with all the 17 factors in the Revised COPE 68 inventory. We also calculate reliability coefficients for all the factors, first-order and second-order, for both the Slovak and English versions.

## 4. Results

### 4.1. Factor analysis of the revised COPE 68 inventory

#### 4.1.1. ESEM analyses, target rotation with the WLSMV

***English sample***. The ESEM results showed an excellent fit of the model with the data, *χ*2(1258) = 1624.585, *p* < 0.001, CFI = 0.992, TLI = 0.986, SRMR = 0.017, and RMSEA = 0.019, 90% CI [0.016, 0.021], and average factor loadings (M = 0.614, see Appendix 1A) ranging from 0.133 to 0.992. Both the fit indices and factor loadings supported the seventeen-dimensional model of the Revised COPE 68 Inventory.

***Slovak sample***. ESEM results showed an excellent fit of the model with the data, *χ*2(1258) = 2225.029, *p* < 0.001, CFI = 0.991, TLI = 0.984, SRMR = 0.014, and RMSEA = 0.023, 90% CI [0.022, 0.025], and average factor loadings (M = 0.617, see Appendix 1B) ranging from 0 to 0.988. Both the fit indices and factor loadings supported the seventeen-dimensional model of the Revised COPE 68 Inventory.

#### 4.1.2. Second-order confirmatory factor analysis and exploratory factor analysis with the COPE inventory scores

In accordance with the analytical procedures utilized in a study by Litman (2006), we evaluated the Revised COPE 68 Inventory scores using iterated principal axis factor analysis with promax rotation allied with the squared multiple correlation for the communality estimate. The factor extraction yielded three factors: Factor 1 representing Active Coping, Factor 2 representing Avoidant Coping, and Factor 3 representing Social Emotional Coping (Tables 1A,B).

**Table 1:** EFA Factor loadings of three-factor model of the revised COPE 68 inventory scores.

The revised COPE 68 Inventory first-order factors	Factor 1	Factor 2	Factor 3
**(A) English sample**			
Positive reinterpretation	**0.715**	−0.066	−0.001
Active coping	**0.742**	−0.143	−0.043
Restraint	**0.505**	0.386	−0.172
Planning	**0.694**	−0.212	−0.021
Acceptance	**0.422**	0.157	−0.042
Suppression of competing activities	**0.575**	0.119	−0.031
Self-care	**0.572**	0.119	0.188
Care for others	**0.388**	0.222	0.089
Use of emotional support	0.005	−0.082	**0.939**
Use of instrumental support	0.225	−0.065	**0.656**
Focus on and venting of emotions	−0.163	0.137	**0.636**
Religion	0.138	**0.220**	0.008
Denial	−0.014	**0.645**	0.038
Behavioural disengagement	−0.171	**0.716**	0.088
Substance use	−0.075	**0.469**	−0.083
Humor	0.177	**0.373**	−0.106
Mental disengagement/Self-distraction	0.203	**0.473**	0.132
**(B) Slovak sample**			
Positive reinterpretation	**0.777**	−0.127	−0.074
Active coping	**0.751**	0.034	−0.164
Restraint	**0.743**	−0.067	0.056
Planning	**0.649**	0.074	−0.173
Acceptance	**0.602**	−0.042	0.154
Suppression of competing activities	**0.478**	0.098	0.120
Self-care	**0.695**	0.072	0.032
Care for others	**0.460**	0.168	0.076
Use of emotional support	−0.052	−0.095	**0.892**
Use of instrumental support	0.209	−0.101	**0.684**
Focus on and venting of emotions	−0.034	0.161	**0.634**
Religion	0.063	0.084	**0.153**
Denial	0.085	**0.640**	−0.030
Behavioural disengagement	−0.141	**0.645**	0.173
Substance use	−0.071	**0.326**	0.015
Humor	0.216	**0.372**	−0.183
Mental disengagement/Self-distraction	0.287	**0.377**	0.045

Factor 1, active coping; Factor 2, avoidant coping; Factor 3, social–emotional coping. Bold = factor loading.

In the English sample, the three factors together explained 39.48% of the variance (Factor 1 = 19.92%, Factor 2 = 11.32%, and Factor 3 = 8.25%). The result of the Kaiser–Meyer–Olkin (KMO) test was 0.791, indicating that the data was well-suited for factor analysis. Moreover, Bartlett’s test of sphericity was significant at *p* < 0.0001, *χ*^2^(136) = 4026.33 (see Table 3A). In the Slovak sample, the three factors together explained 40.56% of the variance (Factor 1 = 25.16%, Factor 2 = 6.14%, and Factor 3 = 9.26%). The result of the KMO test was 0.830, indicating that the data was well-suited for factor analysis. Moreover, Bartlett’s test of sphericity was significant at *p* < 0.0001, *χ*^2^(136) = 7,810, see Table 3B. We performed the EFA with the total score of each of the 17 first-order factors so as to compare the results with the previous studies (e.g., Stowell et al., 2001; Litman, 2006), despite this procedure not being parsimonious and not part of current methodological practices.

We therefore also ran a second-order CFA to simultaneously test the first level (17 first-order factors) and second level (3 s-order factors) structure (see Table 4A for the English sample and Table 4B for the Slovak sample). The second-order CFA model had a good fit with the data in the English sample: *χ*^2^(2190) = 7103.319, *p* < 0.001, CFI = 0.894, TLI = 0.890, SRMR = 0.076 and RMSEA = 0.052, 90% CI [0.051, 0.053]. The factor loadings for the first-order factors on second-order factors are given in Table 2A. The second-order CFA model had a good fit with the data in the Slovak sample: *χ*^2^(2190) = 9282.319, *p* < 0.001, CFI = 0.892, TLI = 0.890, SRMR = 0.078 and RMSEA = 0.056, 90% CI [0.055, 0.057]. The factor loadings for the first-order factors on second-order factors are reported in Table 2B.

**Table 2:** CFA Factor loading of first-order factors on three second-order factors of the Revised COPE 68 inventory.

The Revised COPE 68 Inventory first-order factors	Active coping	Social–emotional coping	Avoidant coping
**(A) English sample**			
Positive reinterpretation	0.842		
Active coping	0.934		
Restraint	0.508		
Acceptance	0.452		
Suppression of competing activities	0.714		
Planning	0.834		
Self-care	0.889		
Care for others	0.423		
Focus on and venting of emotions		0.578	
Use of instrumental support		0.977	
Use of emotional support		0.914	
Mental disengagement			0.421
Denial			0.801
Humor			0.328
Religion			0.198
Behavioral disengagement			0.876
Substance use			0.593
**(B) Slovak sample**			
Positive reinterpretation	0.792		
Active coping	0.980		
Restraint	0.831		
Acceptance	0.635		
Suppression of competing activities	0.643		
Planning	0.854		
Self-care	0.872		
Care for others	0.575		
Focus on and venting of emotions		0.625	
Use of instrumental support		0.999	
Religion		0.192	
Use of emotional support		0.831	
Mental disengagement			0.001
Denial			0.635
Humor			0.116
Behavioral disengagement			0.886
Substance use			0.436

The CFA factor analyses supported the three second-order factors model of the Revised COPE 68 Inventory with the same first-order factors loading in the same first-order factors except for Religion, which loaded into Avoidant Coping in the English version and into Social Emotional Coping in the Slovak version. The new first-order factors *Self-care* and *Care for Others* loaded into the second-order factor Active Coping.

### 4.2. Reliability analyses

Coefficients of reliability for the first-order factors (Cronbach’s alphas) are presented in Table 3. They ranged from 0.53 to 0.93 in the English sample, and from 0.63 to 0.95 in the Slovak sample. We also calculated the Cronbach’s alpha and McDonald’s omega (the composite reliability) for the second-order factors – the Omega total (all explained variance) and the Omega hierarchical (variance explained by a strong single general factor, see Rodriguez et al., 2016). See Table 4.

**Table 3:** Reliability of the first-order factors in Revised COPE 68 Inventory.

COPE first-order factors	1	2	3	4	5	6	7	8	9	10	11	12	13	14	15	16	17
(A) Cronbach’s alpha English sample	0.72	0.65	0.75	0.84	0.67	0.77	0.93	0.86	0.79	0.53	0.87	0.94	0.68	0.54	0.80	0.56	0.75
(B) Cronbach’s alpha Slovak sample	0.75	0.68	0.75	0.84	0.69	0.69	0.95	0.90	0.77	0.63	0.88	0.93	0.73	0.60	0.76	0.68	0.78

1 = Acceptance. 2 = Active coping. 3 = Behavioural disengagement. 4 = Denial. 5 = Use of emotional support. 6 = Humor. 7 = Use of instrumental support. 8 = Mental disengagement. 9 = Planning. 10 = Positive reinterpretation. 11 = Religion. 12 = Restraint. 13 = Substance use. 14 = Suppression of competing activities. 15 = Focus on and venting of emotions. 16 = Self-care. 17 = Care for others.

**Table 4:** Reliability of the second-order factors in Revised COPE 68 Inventory.

COPE second-order factors		Active coping	Social–emotional coping	Avoidant coping
(A) English sample	Cronbach’s alpha	0.86	0.89	0.86
	Omega total	0.90	0.92	0.93
	Omega hierarchical	0.73	0.77	0.70
(B) Slovak sample	Cronbach’s alpha	0.91	0.88	0.81
	Omega total	0.93	0.93	0.90
	Omega hierarchical	0.74	0.70	0.67

## 5. Discussion

The aim of this research study was to develop the Revised COPE 68 Inventory by changing the *Mental Disengagement* items and adding items for the two new first-order factors *Self-care* and *Care for Others* and to test its reliability and perform factor analyses on the first and second-order structure.

The reliability analysis showed improvements in the modified first-order factor *Mental Disengagement* compared to Halamová et al. (2022) as a result of the changes to the formulations and the creation of three new items out of the original four items for *Mental Disengagement* in the COPE inventory (Carver et al., 1989). Consequently, the reliability of *Mental Disengagement* increased from 0.55 to 0.86 and the factor loading improved from values ranging between 0.210 and 0.381 to between 0.531 and 0.881. Changing three items instead of the original four was therefore beneficial.

However, the reliability values for some of the factors decreased, namely *Positive Reinterpretation* (0.53) and *Suppression of Competing Activities* (0.54). Interpretations of Cronbach’s alpha seem rather arbitrary, with different scholars interpreting the coefficient differently (Taber, 2018). Ones with a value of 0.50 are reported as acceptable, satisfactory, and unsatisfactory. The cut-off score is usually 0.70, based on Nunally’s work (1978). However, as Cho and Kim (2015) point out Nunally’s work is not empirically based and nor does it provide a clear explanation of the cut-off level. It was probably only suggested to guide other scholars. They also warn that artificially inflating the coefficient could render it less valid and lead to the so-called attenuation paradox. We therefore chose not to change the number of items to enhance the coefficient. Moreover, a smaller number of items usually contribute to lower internal consistency (Urbánek et al., 2011).

The reliability coefficients are consistent with previous studies that reported lower Cronbach’s alpha for the same first-order factors (e.g., Kallasmaa and Pulver, 2000; Crașovan and Sava, 2013). The reliability coefficient indicates some instability in the first-order factor despite being collected in the same language version. That suggests the results may be sensitive to the characteristics of the sample. For example, the Slovak translation by Halamová et al. (2022) yielded a reliability coefficient of 0.78 for *Positive Reinterpretation* and 0.69 for *Suppression of Competing Activities*. However, the same translation with an additional eight items for the two different first-order factors gave coefficients of 0.53 and 0.54, respectively. In both the study by Halamová et al. (2022) and this study the male–female proportion (67% women in this study compared to 53% in the other) and mean age (33 years, SD = 14.59, in this study compared to 47.16 years, SD = 17.06) differ.

The factor analyses confirmed the first-order factor structure of 15 plus 2 factors of the Revised COPE 68 inventory, which is a new finding. The factor analyses supported three second-order factors in the English and Slovak samples, which is similar to results by other researchers (Stowell et al., 2001; Litman, 2006). We will now discuss the results in more depth and detail.

Both the EFA and CFA factor analyses supported the three second-order factor model of the Revised COPE 68 Inventory with the same first-order factors loading into the same second-order factors, except for *Religion*, which loaded into Avoidant coping in the English version and into Social Emotional Coping in the Slovak version.

Although Krageloh (2011) in his systematic review of Factor Analyses of the Brief COPE found that *Religion* probably loaded together with maladaptive coping strategies when items were used as indicators in the factor analyses and with adaptive coping strategies when the analyses were conducted at first-order level. We found different results for our Slovak and English samples when analyzing them with the same statistical analyses. Hence, the variation in the results of the factor analyses is probably not down to the use of diverse and often inappropriate factor analytic techniques as Krageloh (2011) suggested, but due to the diverse cultural background and role of religion in the culture as Halamová et al. (2022) supposed. *Religion* also had low loadings in all the second-order factors (ranging from 0.153 to 0.220), which are all below the generally accepted level of 0.3. In fact, Religion clustered very poorly in both samples with either avoidant or socially-oriented factor which might be interpreted that Religion does not cluster with any other coping strategies in any sample.

In addition, Humor also loaded in a different factor in present study compared to Stowell et al. (2001) and Litman’s (2006) investigations but similarly to Halamová et al. (2022) or Kowalczuk et al. (2021). However, in the present study we tested factor structure in both Slovak and English samples so it could be hardly attributable just to cultural differences in utilizing humor preferably in negative and self-deprecating in Slovakia as was reported by Halamová et al. (2022). Martin et al. (2003) proposed four types of humor: self-enhancing or self-defeating uses of humor related to self or other directed affiliative or aggressive humor which explain that humor could be used in positive as well as negative ways. This is further explained in terms of humor as coping skills by Doosje et al. (2010). In their interpretation humor could be used to suppress or avoid ongoing negative emotional responses and not to deal with the stress directly and actively which is probably the way how homnour was used by our participants in both Slovak and English samples.

Recently self-care has become a popular coping strategy (Wyatt and Ampadu, 2022) and so it makes sense to include *Self-care* and *Care for Others* in the coping strategies, in addition to the previous 15 factors given in the original COPE measure (Carver et al., 1989). It has also proved relevant in other studies relating to coping (e.g., Eller et al., 2018) and especially pandemic coping (e.g., Ogueji et al., 2022). Similarly, some of the studies relate self-care to self-compassion and care for others to compassion for others, as they show similar attributes (Ogueji and Okoloba, 2020). Based on the results of a longitudinal study, Ironson et al. (2017), pp. 1751 proposed that being compassionate towards oneself as well as others has survival advantages: “*Increasing compassion to the self may also effect a better attitude toward self-care, and thus better health*.” Similarly, Schulman-Green et al. (2016) in their qualitative meta-analysis concluded that experiences of self-care activities or caring activities toward others can positively influence effective control of the person’s mental and physical health via improvements in habits and beliefs. Eller et al. (2018) summarized their concept analysis and concluded that performing self-care activities is associated with positive health outcomes in a diverse population.

The new first-order factors *Self-care* and *Care for Others* loaded into the second-order factor Active Coping which is understandable in terms of these two factors as taking inherently active role in managing stress level.

In our Slovak sample *Self-care* had a lower reliability score than in the English sample, even though the items were identical. Only the language differed and may be attributable to the diverse cultural or social background of the participants. The poor score means that the items are not so much correlated with each other, such as they measure slightly different constructs or different facets of the same constructs, which do not factor together. So, it can be attributable to a presence of a cultural bias or a difficulty in understanding the items. However, it might be possibly related also to the fact, that self-care is uncommon and unknown to Slovaks and so 74% of Slovaks showed symptoms of burnout (Kováč, 2021). Similarly, Pauley and McPherson (2010) reported difficulty in being self-compassionate or self-caring. For some people these activities are demanding, not easy and effortful. Moreover, they may even induce negative experiences such as feelings of anxiety, fear, helplessness, or guilt (Li et al., 2019).

Furthermore, self-care may be linked to care for others. Some people associate self-care with indulgence or selfishness, while caring for others is socially desirable. However, research shows that self-care has numerous benefits such as improved physical health and psychological health, general life satisfaction and work satisfaction including taking care of others (Wyatt and Ampadu, 2022). These two concepts seem to be mutually intertwined, as supportive relationships with others increase self-care (Sebern and Riegel, 2009) and self-care increases care for others (Figley, 2002).

In addition, it is important to emphasize that if it was just the only coping strategy used than Self-care would be considered avoidance. However, by taking care of selves, people decrease their level of emotional arousal which allow them to better cope with their problems. Gunthert et al. (1999) supposed that apart from coping skills it is necessary to consider coping effectiveness as a level of effectiveness of the particular coping strategy to decrease distress and not only to deal with the stress itself. Therefore, self-care could be considered as a form of improving emotion regulation skills which allow further processing the problem (Britton et al., 2012). Similarly, if it was just the only coping strategy used than care for others would be interpreted as avoidance. However, by taking care of others, people usually feel better about themselves and than they are more able to concentrate on solving their own problems by being calmer, having more resources and feeling meaningfulness and security from the social capital they have built. As Gächter et al. (2011) reported the strong correlation between an increased level of social capital and a lower level of stress. Hence, they concluded that stress reduction and coping programs should help to build stronger social networks and social capital.

Lastly, this study has several limitations. Convenience sampling has drawbacks. Also, the online data collection meant only participants with internet access could participate. And lastly, there were twice as many women as men in the sample.

## 6. Conclusion

Both the English and Slovak versions of the Revised COPE 68 inventory were found to be a reliable multidimensional instrument for measuring various coping strategies. The Revised COPE 68 inventory had a good fit for all the 17 first-order factors in both languages. In addition, *Self-care* and *Care for Others* appear to be sound coping strategies that people use for dealing with stressful situations. Although the original studies (Carver et al., 1989) suggested a four higher-order factor solution, a three-factor solution was confirmed in both samples, in the English as well as the Slovak. Also, *Mental Disengagement* was found to function better in the revised version that included more general and up-to-date items.

## Data availability statement

The raw data supporting the conclusions of this article will be made available by the authors, without undue reservation.

## Ethics statement

The studies involving human participants were reviewed and approved by Ethical committee of Faculty of Social and Economic Sciences, Comenius University in Bratislava, Bratislava, Slovakia. The patients/participants provided their written informed consent to participate in this study.

## Author contributions

JH originated the idea of the COPE68. MK performed the statistical analysis. All authors contributed to the article and approved the submitted version.

## Funding

This work was supported by the Slovak Research and Development Agency under the Contract no. PP-COVID-20-0074. Writing this work was supported by the Vedecká grantová agentúra VEGA under grant 1/0075/19.

## Conflict of interest

The authors declare that the research was conducted in the absence of any commercial or financial relationships that could be construed as a potential conflict of interest.

## Publisher’s note

All claims expressed in this article are solely those of the authors and do not necessarily represent those of their affiliated organizations, or those of the publisher, the editors and the reviewers. Any product that may be evaluated in this article, or claim that may be made by its manufacturer, is not guaranteed or endorsed by the publisher.
