# Supplementary material for: The development of the revised COPE 68 inventory with English and Slovak versions
Source: Front Psychol. 2023 Jun 29;14:1202571. doi: 10.3389/fpsyg.2023.1202571 (PMC10344452; doi:10.3389/fpsyg.2023.1202571)
Supplement: Supplementary file 1 [file Data_Sheet_1.docx]

Appendix 1a (English version): Factor loadings of seventeen-factor model of the Revised COPE 68 inventory, ESEM model with the WLSMV

|  | COPE01 | COPE02 | COPE03 | COPE04 | COPE05 | COPE06 | COPE07 | COPE08 | COPE09 | COPE10 | COPE11 | COPE12 | COPE13 | COPE14 | COPE15 | COPE16 | COPE17 |
| --- | --- | --- | --- | --- | --- | --- | --- | --- | --- | --- | --- | --- | --- | --- | --- | --- | --- |
| C05OPE01 | 0.231 | 0.058 | -0.019 | -0.058 | **0.290** | -0.001 | 0.025 | -0.113 | -0.136 | 0.027 | 0.020 | -0.100 | 0.066 | 0.240 | 0.077 | 0.027 | 0.048 |
| C03OPE02 | 0.001 | -0.018 | **0.766** | -0.105 | 0.147 | 0.029 | -0.029 | -0.018 | 0.074 | -0.037 | -0.016 | -0.015 | -0.104 | -0.110 | 0.012 | 0.058 | 0.023 |
| C11OPE03 | 0.084 | 0.011 | 0.184 | 0.210 | 0.048 | -0.065 | 0.012 | -0.038 | 0.051 | -0.038 | **0.601** | -0.038 | -0.007 | -0.003 | 0.081 | -0.006 | -0.053 |
| C08OPE04 | 0.121 | -0.051 | -0.077 | 0.026 | 0.045 | -0.065 | 0.056 | **0.816** | 0.129 | 0.026 | 0.021 | 0.000 | -0.067 | -0.128 | 0.026 | 0.070 | 0.006 |
| C02OPE05 | 0.019 | **0.545** | 0.084 | -0.015 | 0.062 | 0.053 | 0.063 | -0.031 | -0.088 | 0.028 | -0.111 | -0.019 | 0.165 | -0.160 | 0.074 | -0.119 | 0.040 |
| C12OPE06 | -0.016 | 0.013 | 0.013 | 0.026 | 0.004 | -0.077 | 0.002 | 0.013 | -0.040 | 0.010 | -0.021 | **0.967** | 0.010 | -0.033 | -0.012 | 0.012 | 0.002 |
| C01OPE07 | **0.481** | -0.037 | -0.088 | -0.125 | 0.356 | -0.038 | 0.022 | 0.019 | -0.043 | 0.092 | 0.203 | -0.007 | 0.035 | 0.071 | -0.015 | -0.005 | 0.109 |
| C14OPE08 | 0.144 | 0.197 | -0.048 | 0.059 | 0.121 | 0.032 | -0.006 | -0.011 | 0.006 | 0.080 | -0.033 | 0.011 | -0.067 | **0.267** | -0.054 | -0.105 | 0.046 |
| C08OPE09 | -0.009 | 0.009 | 0.055 | 0.054 | 0.063 | -0.044 | -0.046 | **0.867** | -0.050 | -0.009 | -0.064 | 0.010 | 0.030 | -0.041 | 0.008 | -0.036 | 0.014 |
| C13OPE10 | 0.007 | -0.037 | 0.035 | 0.034 | 0.101 | -0.017 | -0.022 | 0.127 | -0.009 | 0.233 | -0.044 | -0.007 | **0.423** | -0.070 | 0.039 | -0.009 | -0.012 |
| C06OPE11 | 0.076 | -0.030 | 0.058 | 0.036 | 0.017 | **0.619** | 0.093 | 0.016 | -0.035 | 0.067 | 0.005 | 0.061 | -0.090 | 0.078 | -0.024 | -0.083 | -0.024 |
| C10OPE12 | 0.190 | 0.021 | 0.021 | 0.035 | 0.008 | -0.038 | 0.033 | 0.024 | 0.105 | **0.598** | -0.109 | -0.094 | -0.190 | 0.040 | 0.106 | -0.029 | 0.030 |
| C01OPE13 | **0.634** | 0.023 | -0.002 | 0.022 | -0.164 | 0.093 | 0.062 | -0.027 | -0.071 | 0.068 | 0.015 | 0.010 | 0.098 | 0.099 | 0.077 | 0.101 | -0.063 |
| C07OPE14 | -0.045 | -0.003 | -0.041 | 0.021 | 0.053 | -0.056 | **0.992** | 0.030 | -0.041 | 0.042 | -0.005 | -0.024 | -0.026 | -0.033 | -0.049 | 0.064 | 0.005 |
| C12OPE15 | 0.022 | 0.013 | -0.007 | -0.023 | 0.002 | 0.035 | 0.008 | 0.035 | -0.023 | -0.039 | 0.030 | **0.952** | 0.046 | 0.037 | 0.021 | -0.058 | 0.009 |
| C14OPE16 | -0.002 | -0.003 | 0.010 | -0.020 | 0.167 | -0.046 | -0.004 | -0.008 | -0.070 | 0.056 | 0.123 | 0.030 | 0.177 | **0.485** | -0.010 | 0.049 | -0.047 |
| C11OPE17 | 0.077 | 0.074 | 0.134 | 0.272 | -0.111 | 0.010 | 0.012 | 0.014 | 0.037 | -0.065 | **0.581** | -0.073 | 0.031 | 0.069 | 0.068 | -0.038 | 0.012 |
| C15OPE18 | 0.050 | -0.033 | -0.020 | 0.029 | 0.100 | -0.027 | 0.028 | 0.061 | -0.009 | 0.052 | 0.107 | -0.017 | -0.041 | -0.002 | **0.638** | 0.017 | 0.012 |
| C03OPE19 | -0.071 | 0.030 | **0.726** | -0.111 | -0.052 | 0.015 | -0.042 | -0.005 | -0.022 | 0.104 | -0.008 | -0.037 | 0.033 | 0.074 | -0.053 | -0.062 | -0.042 |
| C15OPE20 | 0.078 | 0.066 | -0.039 | -0.014 | 0.228 | -0.075 | -0.037 | 0.015 | -0.062 | 0.012 | 0.041 | -0.050 | 0.080 | 0.068 | **0.507** | 0.040 | 0.042 |
| C07OPE21 | -0.025 | 0.031 | -0.035 | -0.010 | -0.002 | 0.016 | **0.986** | 0.008 | -0.019 | -0.013 | 0.037 | -0.037 | 0.031 | -0.018 | 0.012 | -0.047 | 0.022 |
| C10OPE22 | -0.136 | -0.037 | 0.070 | 0.027 | 0.046 | 0.157 | 0.053 | 0.079 | 0.114 | **0.410** | -0.077 | 0.017 | 0.195 | -0.002 | -0.128 | 0.039 | -0.050 |
| C12OPE23 | 0.042 | 0.020 | 0.014 | -0.006 | 0.028 | 0.045 | 0.017 | 0.003 | 0.073 | -0.026 | -0.035 | **0.898** | -0.030 | -0.007 | 0.027 | -0.027 | 0.006 |
| C02OPE24 | 0.008 | **0.331** | 0.082 | -0.043 | -0.103 | 0.109 | -0.012 | 0.061 | -0.088 | 0.202 | 0.069 | 0.187 | -0.015 | 0.128 | -0.015 | 0.039 | 0.025 |
| C14OPE25 | 0.073 | -0.039 | 0.050 | -0.141 | 0.041 | -0.004 | 0.006 | 0.004 | 0.005 | 0.108 | 0.065 | 0.035 | -0.038 | **0.538** | 0.036 | -0.001 | 0.052 |
| C09OPE26 | -0.132 | 0.009 | 0.137 | -0.018 | -0.008 | 0.132 | 0.014 | 0.055 | **0.505** | 0.104 | -0.041 | -0.002 | 0.178 | -0.041 | -0.115 | 0.079 | -0.038 |
| C01OPE27 | **0.566** | -0.054 | -0.022 | -0.077 | -0.168 | 0.083 | 0.048 | 0.078 | -0.059 | 0.017 | 0.057 | -0.040 | 0.088 | 0.029 | 0.142 | 0.233 | 0.028 |
| C10OPE28 | 0.084 | 0.031 | -0.122 | 0.108 | -0.098 | -0.103 | 0.014 | -0.061 | 0.023 | **0.560** | 0.005 | 0.030 | 0.046 | 0.101 | 0.144 | 0.091 | 0.050 |
| C08OPE29 | -0.094 | 0.054 | -0.016 | -0.052 | -0.091 | 0.038 | 0.041 | **0.730** | -0.064 | 0.087 | 0.059 | 0.038 | 0.033 | 0.110 | 0.011 | -0.009 | -0.002 |
| C07OPE30 | 0.040 | -0.018 | 0.055 | 0.012 | -0.006 | -0.036 | **0.859** | -0.005 | 0.107 | 0.018 | -0.056 | 0.047 | -0.045 | -0.014 | 0.038 | 0.002 | 0.018 |
| C17OPE31 | -0.048 | -0.032 | -0.002 | -0.111 | 0.031 | 0.064 | 0.019 | 0.038 | 0.004 | -0.031 | 0.088 | -0.045 | 0.049 | -0.048 | 0.042 | -0.083 | **0.875** |
| C17OPE32 | -0.126 | -0.015 | -0.042 | -0.033 | 0.020 | 0.009 | 0.011 | -0.006 | 0.047 | -0.005 | 0.058 | 0.014 | -0.041 | 0.042 | -0.043 | 0.022 | **0.869** |
| C11OPE33 | 0.003 | -0.083 | 0.065 | 0.186 | -0.111 | 0.138 | -0.004 | -0.032 | -0.006 | -0.078 | **0.471** | 0.023 | -0.006 | -0.010 | 0.069 | 0.231 | 0.253 |
| C16OPE34 | 0.108 | -0.058 | -0.002 | -0.077 | 0.131 | -0.069 | 0.029 | -0.087 | -0.018 | 0.065 | 0.043 | -0.040 | 0.005 | 0.030 | 0.114 | **0.547** | -0.017 |
| C15OPE35 | 0.001 | -0.004 | -0.047 | -0.106 | -0.038 | 0.051 | 0.013 | -0.029 | -0.141 | 0.022 | 0.118 | 0.015 | 0.079 | -0.003 | **0.809** | 0.133 | 0.004 |
| C03OPE36 | 0.038 | -0.020 | **0.652** | 0.050 | 0.019 | -0.062 | 0.061 | 0.018 | 0.006 | -0.037 | 0.202 | -0.046 | -0.005 | -0.041 | 0.067 | 0.063 | -0.006 |
| C13OPE37 | 0.108 | 0.002 | 0.022 | -0.068 | -0.064 | -0.058 | -0.002 | -0.033 | 0.133 | 0.009 | 0.041 | 0.030 | **0.596** | -0.026 | 0.072 | 0.026 | 0.003 |
| C15OPE38 | -0.003 | 0.015 | -0.002 | 0.119 | 0.131 | 0.010 | -0.035 | -0.035 | -0.015 | 0.141 | 0.003 | -0.040 | 0.015 | 0.057 | **0.601** | -0.106 | 0.010 |
| C06OPE39 | -0.002 | 0.057 | 0.049 | -0.085 | 0.070 | **0.792** | 0.026 | -0.012 | 0.031 | -0.078 | 0.117 | 0.026 | -0.067 | 0.069 | 0.020 | -0.016 | 0.028 |
| C02OPE40 | -0.072 | **0.487** | -0.026 | 0.018 | -0.031 | 0.264 | -0.014 | 0.047 | 0.016 | 0.026 | 0.065 | -0.032 | 0.051 | -0.006 | -0.104 | 0.190 | 0.048 |
| C16OPE41 | -0.046 | 0.409 | -0.023 | -0.015 | -0.097 | -0.017 | -0.024 | 0.055 | 0.080 | 0.099 | 0.074 | -0.078 | -0.017 | 0.067 | 0.022 | **0.262** | 0.025 |
| C02OPE42 | -0.066 | **0.856** | -0.031 | 0.032 | 0.041 | -0.070 | 0.009 | 0.005 | 0.115 | -0.098 | 0.005 | 0.018 | -0.057 | -0.030 | 0.048 | 0.144 | 0.016 |
| C07OPE43 | 0.008 | 0.016 | 0.015 | -0.038 | -0.021 | 0.099 | **0.885** | -0.046 | -0.043 | -0.039 | 0.011 | 0.038 | 0.039 | 0.044 | -0.053 | -0.024 | -0.027 |
| C17OPE44 | -0.078 | 0.080 | 0.029 | 0.013 | -0.007 | -0.062 | 0.015 | -0.028 | -0.042 | 0.182 | -0.048 | 0.080 | 0.035 | 0.020 | -0.011 | 0.054 | **0.573** |
| C10OPE45 | 0.015 | -0.003 | 0.032 | 0.060 | -0.158 | 0.140 | -0.025 | 0.006 | 0.089 | **0.223** | -0.141 | 0.014 | 0.116 | 0.041 | 0.129 | 0.004 | 0.185 |
| C06OPE46 | 0.052 | 0.087 | -0.019 | 0.105 | -0.020 | **0.691** | 0.037 | 0.015 | 0.187 | 0.077 | -0.078 | 0.007 | -0.113 | -0.055 | -0.017 | -0.013 | -0.030 |
| C11OPE47 | 0.062 | 0.027 | 0.164 | 0.244 | -0.021 | -0.052 | 0.003 | -0.030 | 0.051 | -0.072 | **0.652** | -0.046 | 0.001 | -0.013 | 0.105 | 0.019 | -0.030 |
| C04OPE48 | 0.023 | 0.044 | 0.016 | **0.596** | 0.005 | 0.015 | 0.000 | 0.004 | 0.058 | 0.005 | 0.343 | -0.060 | -0.017 | 0.017 | -0.035 | 0.018 | 0.036 |
| C05OPE49 | -0.015 | -0.028 | 0.113 | 0.090 | **0.306** | 0.076 | 0.036 | 0.053 | 0.008 | -0.143 | -0.062 | 0.041 | 0.032 | 0.176 | 0.337 | 0.115 | -0.032 |
| C16OPE50 | 0.149 | 0.188 | 0.158 | 0.047 | 0.101 | -0.119 | -0.007 | 0.109 | 0.007 | 0.026 | 0.063 | 0.053 | -0.019 | 0.066 | -0.107 | **0.472** | 0.011 |
| C09OPE51 | -0.059 | -0.003 | -0.019 | -0.070 | -0.073 | 0.049 | 0.039 | 0.030 | **0.733** | 0.040 | 0.090 | 0.030 | 0.067 | 0.076 | -0.043 | -0.063 | -0.009 |
| C04OPE52 | -0.030 | 0.003 | 0.065 | **0.613** | 0.079 | -0.005 | 0.005 | 0.015 | -0.079 | 0.065 | 0.252 | 0.002 | 0.026 | 0.028 | 0.028 | -0.014 | 0.034 |
| C08OPE53 | -0.011 | -0.017 | -0.011 | -0.016 | -0.035 | 0.074 | -0.050 | **0.897** | -0.039 | -0.109 | -0.014 | 0.002 | 0.030 | 0.085 | -0.029 | -0.027 | 0.011 |
| C12OPE54 | -0.012 | -0.015 | -0.046 | 0.038 | -0.001 | 0.004 | -0.004 | 0.006 | 0.065 | 0.017 | -0.015 | **0.910** | -0.031 | -0.013 | 0.000 | 0.055 | 0.001 |
| C05OPE55 | 0.040 | 0.043 | 0.049 | 0.056 | **0.133** | -0.087 | 0.011 | 0.055 | -0.100 | 0.066 | -0.144 | -0.102 | 0.064 | 0.196 | 0.324 | -0.002 | 0.109 |
| C09OPE56 | 0.005 | 0.023 | 0.015 | 0.000 | 0.065 | 0.017 | 0.034 | -0.032 | **0.787** | 0.029 | 0.001 | 0.060 | 0.006 | 0.061 | 0.000 | -0.040 | 0.026 |
| C17OPE57 | 0.330 | 0.057 | 0.135 | 0.120 | -0.082 | -0.088 | 0.008 | 0.053 | -0.019 | -0.057 | -0.147 | -0.002 | -0.044 | -0.015 | -0.016 | -0.008 | **0.511** |
| C03OPE58 | -0.071 | 0.038 | **0.536** | 0.168 | -0.090 | 0.012 | 0.004 | -0.064 | 0.032 | -0.069 | 0.078 | 0.087 | 0.061 | 0.077 | -0.148 | 0.009 | 0.129 |
| C06OPE59 | 0.067 | 0.076 | -0.072 | 0.015 | 0.017 | **0.654** | -0.009 | 0.079 | 0.126 | -0.026 | -0.067 | 0.027 | 0.049 | -0.061 | 0.037 | -0.055 | 0.016 |
| C09OPE60 | -0.024 | 0.024 | 0.033 | -0.070 | -0.007 | 0.206 | -0.037 | 0.002 | **0.451** | 0.083 | 0.084 | 0.137 | 0.073 | -0.047 | -0.030 | 0.117 | 0.012 |
| C16OPE61 | 0.398 | 0.051 | -0.008 | 0.177 | -0.104 | -0.051 | 0.060 | 0.016 | -0.003 | -0.121 | -0.122 | -0.037 | 0.016 | 0.134 | 0.073 | **0.172** | 0.122 |
| C14OPE62 | -0.095 | -0.163 | -0.044 | 0.141 | 0.049 | 0.069 | 0.037 | 0.044 | 0.142 | -0.020 | -0.109 | -0.107 | -0.036 | **0.627** | -0.017 | 0.145 | 0.036 |
| C13OPE63 | 0.110 | 0.124 | -0.005 | -0.038 | -0.005 | -0.007 | -0.033 | 0.036 | 0.070 | -0.057 | 0.009 | 0.014 | **0.622** | 0.018 | -0.100 | 0.025 | 0.060 |
| C04OPE64 | -0.034 | -0.015 | 0.069 | **0.653** | 0.029 | 0.034 | 0.058 | -0.004 | -0.088 | 0.080 | 0.134 | -0.010 | 0.024 | 0.082 | 0.057 | 0.031 | -0.011 |
| C13OPE65 | 0.053 | -0.001 | -0.062 | 0.078 | 0.027 | -0.141 | 0.008 | 0.007 | 0.074 | -0.066 | -0.013 | -0.032 | **0.775** | 0.070 | 0.077 | -0.055 | 0.012 |
| C05OPE66 | -0.035 | -0.035 | 0.010 | 0.307 | **0.251** | 0.057 | -0.002 | -0.031 | -0.032 | -0.066 | -0.286 | 0.038 | 0.054 | 0.191 | 0.269 | 0.161 | -0.012 |
| C04OPE67 | 0.026 | 0.033 | 0.008 | **0.694** | 0.040 | 0.002 | -0.053 | 0.035 | -0.061 | -0.006 | 0.121 | 0.021 | 0.037 | 0.002 | -0.013 | 0.076 | -0.020 |
| C01OPE68 | **0.368** | -0.035 | -0.032 | 0.204 | 0.244 | -0.013 | -0.055 | -0.039 | -0.223 | 0.086 | -0.013 | -0.060 | 0.245 | -0.094 | -0.080 | 0.139 | 0.147 |
| **Average** | **0.512** | **0.555** | **0.670** | **0.639** | **0.245** | **0.689** | **0.931** | **0.828** | **0.619** | **0.448** | **0.576** | **0.932** | **0.604** | **0.479** | **0.639** | **0.363** | **0.707** |

*Notes.* COPE01 = Positive reinterpretation. COPE02 = Mental disengagement. COPE03 = Focus on and venting of emotions. COPE04 = Use of instrumental social support. COPE05 = Active coping. COPE06 = Denial. COPE07 = Religious coping. COPE08 = Humor. COPE09 = Behavioral disengagement. COPE10 = Restraint. COPE11 = Use of emotional social support. COPE12 = Substance use. COPE13 = Acceptance. COPE14 = Suppression of competing activities. COPE15 = Planning. COPE16 = Self-care. COPE17 = Care for others.

Appendix 1b (Slovak version): Factor loadings of seventeen-factor model of the Revised COPE 68 inventory, ESEM model with the WLSMV

|  | COPE01 | COPE02 | COPE03 | COPE04 | COPE05 | COPE06 | COPE07 | COPE08 | COPE09 | COPE10 | COPE11 | COPE12 | COPE13 | COPE14 | COPE15 | COPE16 | COPE17 |
| --- | --- | --- | --- | --- | --- | --- | --- | --- | --- | --- | --- | --- | --- | --- | --- | --- | --- |
| C05OPE01 | 0,135 | -0,057 | 0,104 | 0,075 | 0,107 | -0,020 | 0,020 | -0,005 | -0,066 | 0,188 | -0,021 | -0,026 | -0,034 | 0,103 | 0,247 | 0,026 | 0,037 |
| C03OPE02 | -0,027 | -0,058 | 0,684 | 0,009 | 0,050 | 0,079 | -0,008 | -0,018 | 0,018 | -0,109 | 0,100 | -0,009 | 0,041 | -0,003 | -0,012 | 0,044 | -0,056 |
| C11OPE03 | 0,120 | -0,054 | 0,169 | 0,239 | -0,001 | -0,040 | -0,016 | 0,017 | 0,050 | 0,072 | 0,619 | 0,002 | -0,062 | -0,126 | 0,112 | -0,033 | 0,017 |
| C08OPE04 | 0,068 | 0,024 | 0,101 | -0,005 | 0,062 | 0,041 | -0,005 | 0,842 | -0,065 | -0,028 | -0,039 | -0,035 | -0,031 | -0,032 | 0,006 | -0,060 | 0,033 |
| C02OPE05 | 0,065 | **0,637** | -0,021 | 0,038 | 0,025 | 0,034 | -0,026 | 0,014 | -0,105 | 0,054 | -0,042 | -0,018 | 0,028 | -0,094 | 0,149 | -0,211 | 0,034 |
| C12OPE06 | 0,013 | 0,044 | 0,035 | -0,014 | 0,002 | -0,032 | -0,017 | 0,004 | -0,037 | -0,022 | -0,006 | 0,948 | -0,021 | 0,002 | 0,024 | -0,017 | 0,008 |
| C01OPE07 | 0,281 | -0,013 | 0,055 | -0,015 | -0,005 | 0,033 | -0,057 | 0,032 | -0,148 | 0,176 | -0,045 | -0,014 | 0,035 | -0,049 | 0,184 | 0,022 | 0,155 |
| C14OPE08 | 0,159 | 0,067 | -0,005 | -0,077 | 0,012 | 0,163 | -0,021 | 0,041 | -0,125 | 0,245 | 0,005 | -0,088 | -0,025 | 0,208 | 0,028 | -0,056 | 0,029 |
| C08OPE09 | 0,039 | 0,061 | 0,096 | 0,009 | 0,039 | 0,013 | -0,054 | 0,877 | -0,029 | -0,012 | -0,027 | -0,008 | -0,004 | -0,009 | -0,010 | -0,059 | 0,002 |
| C13OPE10 | 0,100 | 0,036 | 0,057 | -0,011 | 0,058 | -0,019 | -0,006 | 0,139 | 0,031 | 0,156 | -0,012 | -0,009 | 0,550 | -0,042 | -0,010 | -0,142 | -0,051 |
| C06OPE11 | 0,005 | -0,025 | 0,238 | -0,042 | -0,128 | 0,644 | 0,030 | 0,002 | 0,010 | 0,076 | -0,080 | 0,020 | 0,005 | 0,033 | -0,059 | -0,063 | 0,094 |
| C10OPE12 | -0,023 | 0,006 | -0,100 | -0,073 | -0,126 | -0,031 | 0,021 | 0,009 | 0,133 | 0,782 | 0,074 | -0,027 | 0,050 | 0,022 | -0,010 | -0,022 | -0,013 |
| C01OPE13 | 0,783 | 0,027 | -0,087 | -0,043 | 0,088 | -0,022 | 0,031 | 0,030 | 0,044 | -0,006 | 0,095 | -0,025 | 0,060 | 0,060 | 0,005 | 0,089 | -0,006 |
| C07OPE14 | 0,032 | 0,005 | 0,039 | -0,021 | 0,009 | -0,034 | 0,963 | -0,011 | -0,005 | 0,018 | -0,028 | -0,021 | -0,042 | -0,044 | 0,008 | 0,012 | 0,032 |
| C12OPE15 | -0,026 | 0,033 | 0,053 | -0,013 | -0,085 | 0,023 | 0,007 | 0,020 | -0,021 | 0,067 | -0,047 | 0,948 | 0,006 | -0,007 | -0,019 | -0,018 | 0,009 |
| C14OPE16 | 0,019 | -0,076 | 0,121 | 0,035 | 0,032 | -0,030 | -0,026 | -0,017 | 0,105 | 0,032 | 0,017 | 0,067 | -0,031 | 0,660 | -0,024 | -0,033 | -0,010 |
| C11OPE17 | 0,044 | 0,072 | 0,099 | 0,137 | 0,008 | 0,018 | 0,006 | -0,049 | -0,084 | -0,004 | 0,701 | 0,012 | 0,040 | 0,097 | -0,061 | -0,012 | 0,040 |
| C15OPE18 | 0,168 | 0,175 | -0,001 | -0,044 | -0,154 | -0,023 | 0,050 | -0,006 | -0,026 | -0,013 | 0,103 | -0,074 | -0,053 | 0,078 | 0,560 | -0,133 | -0,036 |
| C03OPE19 | -0,085 | 0,098 | 0,461 | -0,039 | 0,102 | -0,135 | -0,015 | -0,071 | 0,249 | 0,029 | 0,093 | 0,011 | 0,102 | 0,183 | 0,114 | -0,047 | 0,039 |
| C15OPE20 | 0,172 | 0,028 | 0,070 | 0,019 | 0,212 | -0,137 | -0,059 | -0,038 | 0,046 | 0,142 | 0,007 | -0,045 | -0,028 | 0,090 | 0,425 | 0,054 | 0,140 |
| C07OPE21 | 0,024 | -0,011 | 0,023 | -0,037 | -0,010 | -0,012 | 0,988 | -0,018 | -0,011 | -0,018 | -0,035 | 0,009 | -0,017 | -0,006 | 0,000 | 0,003 | 0,023 |
| C10OPE22 | 0,021 | 0,019 | 0,032 | 0,051 | 0,314 | -0,061 | 0,107 | 0,052 | -0,093 | 0,250 | -0,015 | 0,099 | 0,126 | 0,063 | 0,052 | 0,129 | 0,053 |
| C12OPE23 | 0,049 | 0,000 | 0,039 | 0,020 | 0,036 | 0,017 | -0,031 | 0,003 | 0,020 | 0,053 | 0,010 | 0,936 | -0,023 | 0,016 | -0,045 | -0,012 | -0,001 |
| C02OPE24 | -0,099 | **0,531** | -0,002 | -0,110 | -0,149 | -0,030 | 0,009 | 0,010 | 0,006 | -0,081 | 0,105 | 0,128 | -0,017 | 0,259 | -0,027 | 0,112 | -0,013 |
| C14OPE25 | 0,077 | 0,029 | -0,014 | -0,017 | 0,005 | 0,041 | -0,053 | -0,050 | -0,076 | 0,075 | -0,043 | -0,025 | 0,011 | 0,561 | 0,042 | 0,060 | 0,032 |
| C09OPE26 | -0,143 | 0,081 | 0,169 | -0,047 | -0,029 | 0,114 | -0,007 | -0,005 | 0,497 | 0,090 | 0,036 | 0,028 | 0,094 | 0,094 | -0,090 | -0,066 | -0,028 |
| C01OPE27 | 0,725 | -0,009 | -0,090 | -0,052 | -0,007 | -0,010 | 0,070 | 0,037 | 0,040 | -0,024 | 0,095 | 0,032 | 0,147 | 0,093 | -0,016 | 0,112 | 0,024 |
| C10OPE28 | 0,082 | 0,020 | -0,158 | 0,019 | -0,036 | 0,009 | 0,007 | -0,031 | -0,064 | 0,592 | 0,074 | 0,001 | 0,056 | 0,069 | -0,023 | 0,107 | 0,022 |
| C08OPE29 | -0,037 | -0,026 | -0,095 | -0,014 | -0,054 | -0,024 | 0,011 | 0,915 | 0,016 | 0,045 | 0,033 | 0,039 | 0,014 | 0,035 | -0,039 | 0,074 | 0,004 |
| C07OPE30 | -0,016 | -0,016 | -0,007 | 0,011 | -0,014 | 0,012 | 0,951 | 0,031 | 0,023 | 0,022 | 0,011 | -0,016 | 0,005 | -0,021 | 0,034 | -0,041 | -0,025 |
| C17OPE31 | -0,029 | -0,018 | -0,023 | -0,079 | 0,004 | 0,024 | 0,011 | 0,029 | 0,009 | -0,028 | 0,097 | -0,004 | -0,034 | -0,044 | -0,006 | -0,042 | 0,910 |
| C17OPE32 | -0,009 | -0,023 | -0,105 | -0,151 | -0,048 | 0,018 | 0,026 | -0,010 | 0,063 | -0,039 | 0,136 | 0,018 | 0,017 | 0,010 | -0,019 | -0,061 | 0,996 |
| C11OPE33 | -0,063 | 0,069 | 0,102 | 0,126 | 0,031 | 0,028 | 0,075 | -0,009 | -0,092 | -0,005 | 0,647 | 0,001 | 0,043 | 0,121 | -0,001 | -0,015 | 0,171 |
| C16OPE34 | 0,067 | 0,014 | 0,019 | -0,079 | -0,024 | 0,045 | -0,058 | 0,043 | -0,153 | 0,154 | 0,129 | -0,038 | 0,033 | 0,035 | 0,144 | 0,444 | -0,072 |
| C15OPE35 | 0,007 | 0,029 | -0,018 | -0,029 | -0,058 | -0,025 | 0,008 | 0,000 | -0,062 | 0,058 | 0,074 | -0,029 | 0,087 | 0,143 | 0,691 | 0,084 | -0,049 |
| C03OPE36 | 0,064 | -0,074 | 0,584 | -0,023 | -0,060 | 0,053 | 0,057 | -0,005 | -0,104 | -0,068 | 0,240 | 0,065 | 0,076 | -0,029 | 0,047 | 0,236 | -0,042 |
| C13OPE37 | 0,000 | -0,025 | 0,118 | -0,138 | 0,068 | 0,036 | -0,059 | -0,007 | 0,073 | 0,012 | 0,034 | -0,021 | 0,619 | -0,101 | 0,132 | 0,062 | 0,005 |
| C15OPE38 | -0,069 | -0,032 | 0,117 | 0,020 | 0,066 | 0,002 | 0,000 | -0,025 | -0,040 | 0,032 | -0,034 | -0,023 | 0,131 | 0,039 | 0,661 | 0,076 | 0,048 |
| C06OPE39 | -0,041 | 0,010 | 0,152 | 0,028 | 0,017 | 0,724 | -0,013 | -0,050 | 0,056 | 0,072 | -0,015 | -0,019 | -0,114 | 0,042 | -0,050 | -0,020 | 0,010 |
| C02OPE40 | -0,056 | **0,554** | -0,040 | -0,052 | 0,040 | 0,120 | 0,054 | 0,026 | 0,070 | 0,035 | 0,102 | -0,026 | 0,071 | -0,114 | -0,068 | 0,180 | -0,039 |
| C16OPE41 | 0,021 | 0,213 | 0,083 | 0,077 | 0,127 | 0,043 | 0,033 | -0,074 | 0,039 | 0,134 | -0,071 | -0,113 | 0,017 | 0,023 | -0,143 | 0,433 | 0,068 |
| C02OPE42 | 0,017 | **0,810** | 0,018 | 0,079 | 0,023 | -0,009 | -0,018 | -0,024 | 0,045 | -0,002 | -0,087 | 0,042 | -0,038 | -0,100 | 0,050 | 0,104 | 0,012 |
| C07OPE43 | -0,015 | 0,012 | -0,026 | 0,017 | 0,014 | 0,020 | 0,900 | -0,027 | 0,020 | 0,028 | 0,020 | 0,005 | 0,006 | 0,016 | -0,034 | -0,016 | -0,018 |
| C17OPE44 | -0,047 | 0,030 | -0,014 | 0,118 | 0,100 | -0,021 | -0,013 | -0,063 | 0,009 | 0,047 | -0,101 | 0,028 | 0,065 | -0,018 | -0,023 | 0,119 | 0,562 |
| C10OPE45 | -0,085 | 0,124 | 0,051 | 0,103 | 0,269 | -0,010 | 0,064 | 0,067 | -0,012 | 0,145 | -0,073 | 0,008 | 0,113 | 0,146 | 0,083 | 0,085 | 0,103 |
| C06OPE46 | -0,040 | 0,065 | -0,140 | 0,036 | -0,002 | 0,746 | 0,018 | 0,014 | 0,046 | -0,089 | 0,075 | 0,101 | 0,037 | 0,005 | 0,022 | 0,050 | -0,051 |
| C11OPE47 | 0,050 | -0,014 | 0,161 | 0,283 | -0,032 | -0,031 | -0,006 | -0,001 | 0,051 | 0,053 | 0,671 | 0,027 | -0,065 | -0,121 | 0,095 | 0,051 | 0,050 |
| C04OPE48 | -0,056 | 0,042 | 0,009 | 0,530 | -0,062 | -0,020 | 0,057 | -0,006 | 0,089 | 0,013 | 0,383 | -0,033 | 0,008 | 0,050 | 0,016 | 0,075 | -0,024 |
| C05OPE49 | -0,026 | -0,019 | -0,084 | 0,112 | -0,091 | 0,041 | -0,013 | -0,011 | 0,057 | 0,120 | -0,050 | -0,005 | 0,018 | 0,035 | 0,270 | 0,335 | 0,149 |
| C16OPE50 | 0,129 | 0,161 | 0,143 | 0,054 | -0,092 | -0,092 | -0,058 | 0,072 | 0,056 | -0,001 | -0,058 | -0,056 | 0,022 | 0,003 | 0,070 | 0,491 | 0,117 |
| C09OPE51 | 0,049 | -0,013 | 0,021 | 0,070 | -0,009 | 0,066 | 0,041 | 0,017 | 0,828 | -0,054 | -0,060 | 0,013 | 0,000 | 0,003 | 0,073 | -0,087 | 0,011 |
| C04OPE52 | -0,006 | 0,014 | 0,028 | 0,595 | 0,066 | 0,031 | -0,036 | 0,011 | -0,038 | 0,005 | 0,288 | -0,057 | 0,000 | 0,021 | 0,010 | 0,055 | 0,099 |
| C08OPE53 | -0,087 | -0,057 | -0,089 | 0,032 | -0,049 | -0,023 | 0,019 | 0,918 | 0,078 | 0,006 | 0,010 | 0,031 | 0,033 | 0,022 | 0,030 | 0,057 | -0,016 |
| C12OPE54 | 0,050 | -0,004 | -0,088 | -0,009 | 0,049 | 0,053 | 0,018 | 0,008 | 0,028 | -0,042 | 0,051 | 0,934 | -0,001 | -0,021 | 0,023 | -0,033 | 0,001 |
| C05OPE55 | 0,029 | -0,033 | -0,048 | 0,152 | 0,037 | 0,022 | 0,049 | -0,029 | -0,137 | 0,095 | -0,094 | -0,061 | 0,113 | 0,064 | 0,372 | -0,026 | 0,104 |
| C09OPE56 | 0,053 | -0,031 | 0,026 | 0,031 | -0,041 | 0,107 | 0,005 | -0,017 | 0,839 | 0,045 | -0,061 | 0,036 | -0,030 | -0,004 | 0,020 | -0,043 | 0,037 |
| C17OPE57 | 0,131 | 0,049 | 0,126 | 0,187 | -0,090 | 0,007 | 0,019 | 0,089 | -0,086 | 0,012 | -0,084 | -0,049 | -0,025 | 0,039 | -0,082 | 0,059 | 0,489 |
| C03OPE58 | -0,142 | 0,091 | 0,591 | 0,037 | -0,053 | 0,006 | 0,071 | 0,000 | 0,150 | -0,083 | 0,115 | 0,039 | 0,048 | 0,088 | 0,013 | -0,026 | 0,047 |
| C06OPE59 | 0,089 | 0,047 | -0,264 | -0,007 | 0,144 | 0,522 | -0,040 | 0,090 | 0,184 | -0,120 | -0,035 | 0,007 | 0,070 | 0,016 | 0,043 | 0,030 | 0,005 |
| C09OPE60 | -0,005 | 0,048 | 0,036 | -0,013 | 0,069 | 0,100 | 0,051 | 0,041 | 0,579 | -0,008 | 0,016 | 0,005 | 0,033 | -0,035 | -0,067 | 0,164 | -0,026 |
| C16OPE61 | 0,368 | 0,103 | 0,063 | 0,115 | -0,132 | 0,033 | 0,073 | 0,029 | -0,141 | 0,006 | -0,048 | -0,078 | 0,023 | 0,065 | -0,024 | 0,257 | 0,084 |
| C14OPE62 | -0,022 | -0,038 | 0,019 | 0,148 | -0,041 | 0,062 | 0,036 | 0,071 | 0,038 | -0,051 | -0,058 | -0,006 | -0,002 | 0,583 | 0,168 | 0,016 | -0,016 |
| C13OPE63 | 0,014 | 0,019 | -0,041 | 0,021 | -0,057 | 0,110 | 0,033 | -0,038 | -0,017 | -0,056 | 0,040 | -0,036 | 0,782 | 0,044 | -0,049 | -0,034 | 0,034 |
| C04OPE64 | 0,028 | 0,008 | -0,041 | 0,716 | 0,021 | -0,026 | 0,041 | 0,003 | -0,016 | 0,037 | 0,114 | 0,011 | 0,058 | 0,077 | -0,026 | -0,015 | 0,025 |
| C13OPE65 | 0,113 | 0,009 | 0,043 | 0,105 | -0,088 | -0,170 | -0,034 | -0,008 | -0,007 | 0,025 | -0,094 | 0,011 | 0,680 | 0,005 | -0,091 | 0,015 | 0,031 |
| C05OPE66 | 0,083 | -0,016 | 0,028 | 0,351 | -0,058 | 0,029 | 0,025 | -0,063 | -0,145 | 0,133 | -0,193 | 0,037 | 0,140 | 0,083 | 0,269 | 0,114 | 0,022 |
| C04OPE67 | 0,011 | 0,021 | 0,038 | 0,723 | -0,053 | -0,009 | -0,027 | 0,006 | 0,021 | -0,002 | 0,074 | 0,003 | 0,059 | 0,067 | -0,042 | 0,022 | 0,017 |
| C01OPE68 | 0,264 | 0,008 | 0,059 | 0,154 | -0,175 | -0,075 | -0,019 | -0,030 | -0,107 | 0,104 | -0,122 | 0,060 | 0,210 | -0,038 | 0,115 | 0,095 | 0,106 |
| **Average** | **0.513** | **0.633** | **0.580** | **0.641** | **0.000** | **0.659** | **0.951** | **0.888** | **0.686** | **0.442** | **0.660** | **0.942** | **0.658** | **0.503** | **0.584** | **0.406** | **0.739** |

*Notes.* COPE01 = Positive reinterpretation. COPE02 = Mental disengagement. COPE03 = Focus on and venting of emotions. COPE04 = Use of instrumental social support. COPE05 = Active coping. COPE06 = Denial. COPE07 = Religious coping. COPE08 = Humor. COPE09 = Behavioral disengagement. COPE10 = Restraint. COPE11 = Use of emotional social support. COPE12 = Substance use. COPE13 = Acceptance. COPE14 = Suppression of competing activities. COPE15 = Planning. COPE16 = Self-care. COPE17 = Care for others.

Appendix 2: Original the COPE 60 inventory and newly developed items of the COPE 68 inventory

# **Mental disengagement**

***Original items of the COPE 60 inventory Mental disengagement***

2. I turn to work or other substitute activities to take my mind off things./ Venujem sa práci alebo iným činnostiam, aby som prestal/a myslieť na veci.

16. I daydream about things other than this./ Cez deň snívam o iných veciach, než je táto.

31. I sleep more than usual./ Spávam dlhšie než zvyčajne.

43. I go to movies or watch TV, to think about it less./ Idem do kina alebo pozerám TV, aby som na to menej myslel/a.

***Newly developed items in the COPE 68 Inventory for Mental Disengagement***

5. I turn to work or other substitute activities to take my mind off things./ Venujem sa práci alebo iným činnostiam, aby som prestal/a myslieť na veci. UNCHANGED

24. I change my environment to avoid thinking about the problem. / Mením prostredie, aby som sa vyhol/la mysleniu na problém. CHANGED

40. I try to think about other things than the problem./ Snažím sa myslieť na iné veci ako na problém. CHANGED

42. I do various activities to think about it less./ Robím rôzne aktivity, aby som na to myslel/a menej. CHANGED

# **Self-care**

***Newly developed items in the COPE 68 Inventory for Self-care***

34. I take care of my needs. / Starám sa o svoje potreby.

41. I try to avoid what makes me feel bad. / Snažím sa vyhýbať veciam, ktoré mi robia zle.

50. I try to seek out what makes me feel good. / Usilujem sa vyhľadávať veci, ktoré mi robia dobre.

61. I encourage myself by thinking about positive things in my life or my previous accomplishments. / Povzbudzujem sa tým, že myslím na pozitívne veci v mojom živote alebo svoje predchádzajúce úspechy.

# **Care for Others**

***Newly developed items in the COPE 68 Inventory for Care for Others***

31. I take care of other people’s needs. / Starám sa o potreby druhých ľudí.

44. I try to avoid what makes other people feel bad. / Snažím sa vyhýbať tomu, čo robí zle iným ľuďom.

32. I seek to do what makes other people feel good. / Usilujem sa robiť to, čo robí dobre iným ľuďom.

57. I encourage other people by reminding them about positive things in their lives or their previous accomplishments. / Povzbudzujem druhých tým, že im pripomínam pozitívne veci o ich živote alebo ich predchádzajúce úspechy.

Appendix 3: English version of the COPE 68

**The COPE 68 Inventory – English version**

We are interested in how people respond when they confront difficult or stressful events in their lives. There are lots of ways to try to deal with stress. This questionnaire asks you to indicate what you generally do and feel when you experience stressful events. Obviously, different events bring out somewhat different responses but think about what you usually do when you are under a lot of stress. Then respond to each of the following items by ticking one possibility. Please try to respond to each item separately in your mind from each other item. Choose your answers thoughtfully, and make your answers as true FOR YOU as you can. Please answer every item. There are no "right" or "wrong" answers, so choose the most accurate answer for YOU-not what you think "most people" would say or do. **INDICATE WHAT YOU USUALLY DO WHEN YOU EXPERIENCE A STRESSFUL EVENT**.

|  | I usually don't do this at all | I usually do this a little bit | I usually do this a medium amount | I usually do this a lot |
| --- | --- | --- | --- | --- |
| 1. I concentrate my efforts on doing something about it. | ❏ | ❏ | ❏ | ❏ |
| 2. I get upset and let my emotions out. | ❏ | ❏ | ❏ | ❏ |
| 3. I discuss my feelings with someone. | ❏ | ❏ | ❏ | ❏ |
| 4. I laugh about the situation. | ❏ | ❏ | ❏ | ❏ |
| 5. I turn to work or other substitute activities to take my mind off things. | ❏ | ❏ | ❏ | ❏ |
| 6. I use alcohol or drugs to make myself feel better. | ❏ | ❏ | ❏ | ❏ |
| 7. I try to grow as a person as a result of the experience. | ❏ | ❏ | ❏ | ❏ |
| 8. I keep myself from getting distracted by other thoughts or activities. | ❏ | ❏ | ❏ | ❏ |
| 9. I make jokes about it. | ❏ | ❏ | ❏ | ❏ |
| 10. I get used to the idea that it happened. | ❏ | ❏ | ❏ | ❏ |
| 11. I say to myself "this isn't real." | ❏ | ❏ | ❏ | ❏ |
| 12. I restrain myself from doing anything too quickly. | ❏ | ❏ | ❏ | ❏ |
| 13. I try to see it in a different light, to make it seem more positive. | ❏ | ❏ | ❏ | ❏ |
| 14. I put my trust in God. | ❏ | ❏ | ❏ | ❏ |
| 15. I drink alcohol or take drugs, in order to think about it less. | ❏ | ❏ | ❏ | ❏ |
| 16. I focus on dealing with this problem, and if necessary let other things slide a little. | ❏ | ❏ | ❏ | ❏ |
| 17. I try to get emotional support from friends or relatives. | ❏ | ❏ | ❏ | ❏ |
| 18. I make a plan of action. | ❏ | ❏ | ❏ | ❏ |
| 19. I get upset, and am really aware of it. | ❏ | ❏ | ❏ | ❏ |
| 20. I think about how I might best handle the problem. | ❏ | ❏ | ❏ | ❏ |
| 21. I seek God's help. | ❏ | ❏ | ❏ | ❏ |
| 22. I hold off doing anything about it until the situation permits. | ❏ | ❏ | ❏ | ❏ |
| 23. I try to lose myself for a while by drinking alcohol or taking drugs. | ❏ | ❏ | ❏ | ❏ |
| 24. I change my environment to avoid thinking about the problem. | ❏ | ❏ | ❏ | ❏ |
| 25. I try hard to prevent other things from interfering with my efforts at dealing with this. | ❏ | ❏ | ❏ | ❏ |
| 26. I admit to myself that I can't deal with it, and quit trying. | ❏ | ❏ | ❏ | ❏ |
| 27. I look for something good in what is happening. | ❏ | ❏ | ❏ | ❏ |
| 28. I make sure not to make matters worse by acting too soon. | ❏ | ❏ | ❏ | ❏ |
| 29. I kid around about it. | ❏ | ❏ | ❏ | ❏ |
| 30. I try to find comfort in my religion. | ❏ | ❏ | ❏ | ❏ |
| 31. I take care of other people´s needs. | ❏ | ❏ | ❏ | ❏ |
| 32. I seek to do what makes other people feel good. | ❏ | ❏ | ❏ | ❏ |
| 33. I get sympathy and understanding from someone. | ❏ | ❏ | ❏ | ❏ |
| 34. I take care of my needs. | ❏ | ❏ | ❏ | ❏ |
| 35. I try to come up with a strategy about what to do. | ❏ | ❏ | ❏ | ❏ |
| 36. I let my feelings out. | ❏ | ❏ | ❏ | ❏ |
| 37. I accept that this has happened and that it can't be changed. | ❏ | ❏ | ❏ | ❏ |
| 38. I think hard about what steps to take. | ❏ | ❏ | ❏ | ❏ |
| 39. I refuse to believe that it has happened. | ❏ | ❏ | ❏ | ❏ |
| 40. I try to think about other things than the problem. | ❏ | ❏ | ❏ | ❏ |
| 41. I try to avoid what makes me feel bad. | ❏ | ❏ | ❏ | ❏ |
| 42. I do various activities to think about it less. | ❏ | ❏ | ❏ | ❏ |
| 43. I pray more than usual. | ❏ | ❏ | ❏ | ❏ |
| 44. I try to avoid what makes other people feel bad. | ❏ | ❏ | ❏ | ❏ |
| 45. I force myself to wait for the right time to do something. | ❏ | ❏ | ❏ | ❏ |
| 46. I pretend that it hasn't really happened. | ❏ | ❏ | ❏ | ❏ |
| 47. I talk to someone about how I feel. | ❏ | ❏ | ❏ | ❏ |
| 48. I try to get advice from someone about what to do. | ❏ | ❏ | ❏ | ❏ |
| 49. I take direct action to get around the problem. | ❏ | ❏ | ❏ | ❏ |
| 50. I try to seek out what makes me feel good. | ❏ | ❏ | ❏ | ❏ |
| 51. I just give up trying to reach my goal. | ❏ | ❏ | ❏ | ❏ |
| 52. I talk to someone to find out more about the situation. | ❏ | ❏ | ❏ | ❏ |
| 53. I make fun of the situation. | ❏ | ❏ | ❏ | ❏ |
| 54. I use alcohol or drugs to help me get through it. | ❏ | ❏ | ❏ | ❏ |
| 55. I do what has to be done, one step at a time. | ❏ | ❏ | ❏ | ❏ |
| 56. I give up the attempt to get what I want. | ❏ | ❏ | ❏ | ❏ |
| 57. I encourage other people by reminding them about positive things in their lives or their previous accomplishments. | ❏ | ❏ | ❏ | ❏ |
| 58. I feel a lot of emotional distress and I find myself expressing those feelings a lot. | ❏ | ❏ | ❏ | ❏ |
| 59. I act as though it hasn't even happened. | ❏ | ❏ | ❏ | ❏ |
| 60. I reduce the amount of effort I'm putting into solving the problem. | ❏ | ❏ | ❏ | ❏ |
| 61. I encourage myself by thinking about positive things in my life or my previous accomplishments. | ❏ | ❏ | ❏ | ❏ |
| 62. I put aside other activities in order to concentrate on this. | ❏ | ❏ | ❏ | ❏ |
| 63. I learn to live with it. | ❏ | ❏ | ❏ | ❏ |
| 64. I talk to someone who could do something concrete about the problem. | ❏ | ❏ | ❏ | ❏ |
| 65. I accept the reality of the fact that it happened. | ❏ | ❏ | ❏ | ❏ |
| 66. I take additional action to try to get rid of the problem. | ❏ | ❏ | ❏ | ❏ |
| 67. I ask people who have had similar experiences what they did. | ❏ | ❏ | ❏ | ❏ |
| 68. I learn something from the experience. | ❏ | ❏ | ❏ | ❏ |

**Scoring:**

1. Positive reinterpretation: 7, 13, 27, 68
2. Mental disengagement: 5, 24, 40, 42
3. Focus on and venting of emotions: 2, 19, 36, 58
4. Use of instrumental social support: 48, 52, 64, 67
5. Active coping: 1, 66, 49, 55
6. Denial: 11, 39, 46, 59
7. Religious coping: 14, 21, 30, 43
8. Humor: 4, 9, 29, 53
9. Behavioral disengagement: 26, 51, 56, 60
10. Restraint: 12, 22, 28, 45
11. Use of emotional social support: 3, 17, 33, 47
12. Substance use: 6, 23, 15, 54
13. Acceptance: 10, 37, 65, 63
14. Suppression of competing activities: 8, 16, 25, 62
15. Planning: 18, 35, 20, 38
16. Self-care 34, 41, 50, 61
17. Care for others 31, 44, 32, 57

Appendix 4: Slovak version of the COPE 68

**The COPE 68 Inventory – Slovak version**

Zaujíma nás, ako ľudia reagujú, keď čelia zložitým alebo stresujúcim udalostiam vo svojom živote. Existuje veľa spôsobov, ako sa pokúsiť vyrovnať sa so stresom. Cieľom tohto dotazníka je, aby ste naznačili, čo obvykle robíte a cítite, keď sa u Vás vyskytnú stresujúce udalosti. Je zrejmé, že rôzne udalosti prinesú trochu odlišné reakcie, ale myslite na to, čo zvyčajne robíte, keď ste pod veľkým stresom. Pokúste sa odpovedať na každú položku osobitne vo svojej mysli nezávisle od každej inej položky. Vyberte si odpovede premyslene, aby boli pre VÁS čo najpravdivejšie. Odpovedzte na každú položku. Neexistujú žiadne „správne“ alebo „nesprávne“ odpovede, preto si vyberte čo najpresnejšiu odpoveď pre VÁS a nie to, čo si myslíte, že by „väčšina ľudí“ povedala alebo urobila. Uveďte, čo obvykle robíte, keď VY zažívate stresujúcu udalosť.

|  | obvykle to nerobím vôbec | zvyčajne to robím trochu | zvyčajne to robím stredne veľa | zvyčajne to robím veľmi veľa |
| --- | --- | --- | --- | --- |
| 1. Usilujem sa s tým niečo urobiť. | ❏ | ❏ | ❏ | ❏ |
| 2. Som rozrušený/á a nechám svoje emócie prejaviť sa. | ❏ | ❏ | ❏ | ❏ |
| 3. Diskutujem s niekým o svojich pocitoch. | ❏ | ❏ | ❏ | ❏ |
| 4. Smejem sa na situácii. | ❏ | ❏ | ❏ | ❏ |
| 5. Venujem sa práci alebo iným činnostiam, aby som prestal/a myslieť na veci. | ❏ | ❏ | ❏ | ❏ |
| 6. Pijem alkohol alebo užívam drogy, aby som sa cítil/a lepšie. | ❏ | ❏ | ❏ | ❏ |
| 7. Usilujem sa osobnostne rásť na základe vlastných skúseností. | ❏ | ❏ | ❏ | ❏ |
| 8. Usilujem sa, aby ma iné myšlienky alebo činnosti nevyrušovali. | ❏ | ❏ | ❏ | ❏ |
| 9. Vtipkujem o tom. | ❏ | ❏ | ❏ | ❏ |
| 10. Zvykám si na myšlienku, že sa to stalo. | ❏ | ❏ | ❏ | ❏ |
| 11. Hovorím si: „toto nie je skutočné“. | ❏ | ❏ | ❏ | ❏ |
| 12. Ovládam sa, aby som niečo neurobil/a príliš rýchlo. | ❏ | ❏ | ❏ | ❏ |
| 13. Snažím sa vidieť to v inom, pozitívnejšom svetle. | ❏ | ❏ | ❏ | ❏ |
| 14. Svoju dôveru vkladám v Boha. | ❏ | ❏ | ❏ | ❏ |
| 15. Pijem alkohol, alebo užívam drogy, aby som na to menej myslel/a. | ❏ | ❏ | ❏ | ❏ |
| 16. Ak je to nutné, odkladám ostatné veci, aby som sa vysporiadal/a s týmto problémom. | ❏ | ❏ | ❏ | ❏ |
| 17. Snažím sa získať citovú oporu priateľov alebo príbuzných. | ❏ | ❏ | ❏ | ❏ |
| 18. Robím si plán činností. | ❏ | ❏ | ❏ | ❏ |
| 19. Som rozrušený/á a skutočne si to uvedomujem. | ❏ | ❏ | ❏ | ❏ |
| 20. Rozmýšľam nad tým, ako by som mohol/la problém čo najlepšie vyriešiť. | ❏ | ❏ | ❏ | ❏ |
| 21. Hľadám pomoc u Boha. | ❏ | ❏ | ❏ | ❏ |
| 22. Riešim to vtedy, keď to situácia umožní. | ❏ | ❏ | ❏ | ❏ |
| 23. Pokúšam sa uvoľniť na chvíľu pitím alkoholu alebo užívaním drog. | ❏ | ❏ | ❏ | ❏ |
| 24. Mením prostredie, aby som sa vyhol/la mysleniu na problém. | ❏ | ❏ | ❏ | ❏ |
| 25. Usilujem sa, aby mi do riešenia tohto problému nezasahovali iné veci. | ❏ | ❏ | ❏ | ❏ |
| 26. Priznávam si, že sa s tým neviem vyrovnať a prestávam sa snažiť. | ❏ | ❏ | ❏ | ❏ |
| 27. Hľadám niečo dobré v tom, čo sa deje. | ❏ | ❏ | ❏ | ❏ |
| 28. Zabezpečím, aby sa nič nezhoršilo príliš rýchlym konaním. | ❏ | ❏ | ❏ | ❏ |
| 29. Zabávam sa na tom. | ❏ | ❏ | ❏ | ❏ |
| 30. Snažím sa nájsť útechu vo svojom náboženstve. | ❏ | ❏ | ❏ | ❏ |
| 31. Starám sa o potreby druhých ľudí. | ❏ | ❏ | ❏ | ❏ |
| 32. Usilujem sa robiť to, čo robí dobre iným ľuďom. | ❏ | ❏ | ❏ | ❏ |
| 33. Hľadám u niekoho spriaznenosť a porozumenie. | ❏ | ❏ | ❏ | ❏ |
| 34. Starám sa o svoje potreby. | ❏ | ❏ | ❏ | ❏ |
| 35. Pokúšam sa vymyslieť si stratégiu, čo robiť. | ❏ | ❏ | ❏ | ❏ |
| 36. Dávam priechod svojim pocitom. | ❏ | ❏ | ❏ | ❏ |
| 37. Uznávam, že sa to stalo a že sa to nedá zmeniť. | ❏ | ❏ | ❏ | ❏ |
| 38. Veľa myslím na to, aké kroky podniknúť. | ❏ | ❏ | ❏ | ❏ |
| 39. Odmietam uveriť, že sa to stalo. | ❏ | ❏ | ❏ | ❏ |
| 40. Snažím sa myslieť na iné veci ako na problém. | ❏ | ❏ | ❏ | ❏ |
| 41. Snažím sa vyhýbať veciam, ktoré mi robia zle. | ❏ | ❏ | ❏ | ❏ |
| 42. Robím rôzne aktivity, aby som na to myslel/a menej. | ❏ | ❏ | ❏ | ❏ |
| 43. Modlím sa viac ako zvyčajne. | ❏ | ❏ | ❏ | ❏ |
| 44. Snažím sa vyhýbať tomu, čo robí zle iným ľuďom. | ❏ | ❏ | ❏ | ❏ |
| 45. Prinútim sa urobiť to, keď na to nastane vhodný čas. | ❏ | ❏ | ❏ | ❏ |
| 46. Predstieram, že sa to v skutočnosti nestalo. | ❏ | ❏ | ❏ | ❏ |
| 47. Rozprávam sa s niekým o tom, ako sa cítim. | ❏ | ❏ | ❏ | ❏ |
| 48. Pokúšam sa získať od niekoho radu, čo robiť. | ❏ | ❏ | ❏ | ❏ |
| 49. Podnikám priame kroky, aby som sa vyhol/la problému. | ❏ | ❏ | ❏ | ❏ |
| 50. Usilujem sa vyhľadávať veci, ktoré mi robia dobre. | ❏ | ❏ | ❏ | ❏ |
| 51. Vzdávam sa už dosiahnutia svojho cieľa. | ❏ | ❏ | ❏ | ❏ |
| 52. Rozprávam sa s niekým, aby som sa o situácii dozvedel/a viac. | ❏ | ❏ | ❏ | ❏ |
| 53. Z tejto situácie si robím srandu. | ❏ | ❏ | ❏ | ❏ |
| 54. Užívam alkohol alebo drogy, aby mi pomohli dostať sa cez to. | ❏ | ❏ | ❏ | ❏ |
| 55. Krok za krokom robím, čo musí byť urobené. | ❏ | ❏ | ❏ | ❏ |
| 56. Vzdávam sa pokusu dosiahnuť, čo chcem. | ❏ | ❏ | ❏ | ❏ |
| 57. Povzbudzujem druhých tým, že im pripomínam pozitívne veci o ich živote alebo ich predchádzajúce úspechy. | ❏ | ❏ | ❏ | ❏ |
| 58. Cítim sa citovo veľmi rozrušený/á a uvedomujem si, že prejavujem veľa týchto svojich pocitov. | ❏ | ❏ | ❏ | ❏ |
| 59. Správam sa, akoby sa to vôbec nestalo. | ❏ | ❏ | ❏ | ❏ |
| 60. Znižujem úsilie, ktoré vynakladám na riešenie tohto problému. | ❏ | ❏ | ❏ | ❏ |
| 61. Povzbudzujem sa tým, že myslím na pozitívne veci v mojom živote alebo svoje predchádzajúce úspechy. | ❏ | ❏ | ❏ | ❏ |
| 62. Odkladám stranou iné činnosti, aby som sa mohol/la na to sústrediť. | ❏ | ❏ | ❏ | ❏ |
| 63. Učím sa s tým žiť. | ❏ | ❏ | ❏ | ❏ |
| 64. Rozprávam sa s človekom, ktorý môže urobiť niečo konkrétne s týmto problémom. | ❏ | ❏ | ❏ | ❏ |
| 65. Prijímam skutočnosť, že sa to stalo. | ❏ | ❏ | ❏ | ❏ |
| 66. Podnikám ďalšie kroky, ktoré mi pomôžu zbaviť sa problému. | ❏ | ❏ | ❏ | ❏ |
| 67. Pýtam sa ľudí s podobnou skúsenosťou, čo oni vtedy robili. | ❏ | ❏ | ❏ | ❏ |
| 68. Z každej skúsenosti sa niečo naučím. | ❏ | ❏ | ❏ | ❏ |

**Scoring:**

1. Pozitívna reinterpretácia: 7, 13, 27, 68
2. Mentálne odpojenie: 5, 24, 40, 42
3. Zameranie na emócie a ich ventilovanie: 2, 19, 36, 58
4. Využitie inštrumentálnej sociálnej opory: 48, 52, 64, 67
5. Aktívne zvládanie: 1, 66, 49, 55
6. Popretie: 11, 39, 46, 59
7. Náboženské zvládanie: 14, 21, 30, 43
8. Humor: 4, 9, 29, 53
9. Behaviorálne odpútanie: 26, 51, 56, 60
10. Zdržiavanie sa: 12, 22, 28, 45
11. Využitie emocionálnej sociálnej opory: 3, 17, 33, 47
12. Užívanie omamných látok: 6, 23, 15, 54
13. Akceptácia: 10, 37, 65, 63
14. Potlačenie súťaživých aktivít: 8, 16, 25, 62
15. Plánovanie: 18, 35, 20, 38
16. Starostlivosť o seba: 34, 41, 50, 61
17. Starostlivosť o druhých: 31, 44, 32, 57
